# Volumetric Analysis of Navigation-Guided Orbital Decompression in Graves’ Orbitopathy: A Case Report

**DOI:** 10.3390/life15081277

**Published:** 2025-08-12

**Authors:** Gonzalo Ruiz-de-Leon, Santiago Ochandiano, Sara Alvarez-Mokthari, Marta Benito-Anguita, Ismael Nieva-Pascual, Pilar Cifuentes-Canorea, Guillermo Sanjuan-de-Moreta, Jose-Ignacio Salmeron, Ignacio Navarro-Cuellar, Carlos Navarro-Cuellar, Manuel Tousidonis

**Affiliations:** 1 Department of Oral and Maxillofacial Surgery, Gregorio Marañon University Hospital, 28007 Madrid, Spain; ruizdeleong@gmail.com (G.R.-d.-L.); saramokthari@gmail.com (S.A.-M.); cnavarrocuellar@gmail.com (C.N.-C.); 2 Gregorio Marañón Research Institute, 28007 Madrid, Spain; 3 Department of Ophtalmology, Gregorio Marañon University Hospital, 28007 Madrid, Spain; 4 Department of Otolaryngology, Gregorio Marañon University Hospital, 28007 Madrid, Spain

**Keywords:** Graves’ orbitopathy, orbital decompression surgery, image guided surgery, volumetric analysis, endoscopic surgery, orbital volume expansion

## Abstract

Graves’ orbitopathy (GO) is a debilitating autoimmune disorder that may require surgical orbital decompression in severe cases with risk of proptosis and optic neuropathy. This report presents a case treated with navigation-assisted three-wall orbital decompression, planned with preoperative imaging and assessed using postoperative analysis. Intraoperative navigation enabled precise localization of critical structures, improving osteotomy execution. Postoperatively, orbital volume increased by 3.5 cm^3^ (right eye) and 4.0 cm^3^ (left eye), while proptosis was reduced by 6 mm in both eyes. These changes correlated with intraocular pressure normalization and functional improvement. This was further supported by a postoperative Clinical Activity Score (CAS) of 0, indicating active orbital inflammation. Image-guided surgery (IGS) achieved an average proptosis reduction of 3.8 mm, slightly superior to that of non-guided techniques. Although IGS enhances precision and functional outcomes, it requires longer surgical time and incurs higher costs, highlighting the need for prospective studies on long-term efficacy This case supports the importance of integrating advanced imaging and navigation-assisted techniques in GO management to improve both functional and aesthetic outcomes.

## 1. Introduction

Graves’ orbitopathy (GO) is the most common extrathyroidal manifestation of Graves’ disease, affecting 25% to 50% of patients with thyroid dysfunction. This autoimmune orbital disorder is characterized by expansion of the orbital contents—primarily through extraocular muscle enlargement and orbital fat hypertrophy—leading to eyelid retraction, proptosis, diplopia, and, in severe cases, compressive optic neuropathy. These manifestations impair both visual function and facial appearance, with significant psychological and social repercussions that diminish quality of life [1,2,3].

Management of moderate to severe GO requires a multidisciplinary approach combining medical therapy in the active phase with surgical intervention during the inactive phase. Orbital decompression is the mainstay surgical treatment, particularly in cases of progressive exophthalmos or optic nerve compromise [4,5,6]. The procedure aims to relieve intra-orbital pressure by removing orbital walls and/or fat, restoring anatomical balance and improving both function and aesthetics.

Conventional decompression techniques rely heavily on the surgeon’s experience and anatomical landmarks, which may limit precision and reproducibility. In this context, image-guided surgery (IGS) has emerged as a valuable tool, enabling individualized 3D preoperative planning and real-time intraoperative navigation. This improves accuracy, especially near critical structures such as the medial wall and optic nerve.

Despite its promise, evidence linking IGS to objective improvements in orbital volume and functional outcomes remains limited. We present a case of severe GO treated with navigation-assisted three-wall decompression, integrating high-resolution CT planning and postoperative volumetric analysis. This report discusses diagnostic workup, surgical strategy, and postoperative evolution, underscoring the potential of IGS to enhance precision and outcomes in complex orbital surgery.

This case was conducted in accordance with the Declaration of Helsinki. Written informed consent was obtained from the patient for publication of this report and accompanying images.

## 2. Case

**Figure 1:**
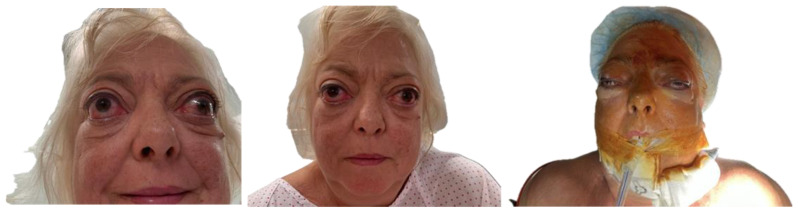
Typical clinical signs of Graves’ orbitopathy. Visible findings include bilateral proptosis, upper eyelid retraction, periorbital edema, conjunctival injection, and restricted extraocular movements, most commonly affecting upward and lateral gaze.

A 54-year-old woman with a significant medical history, including hepatopathy, diabetes mellitus, cervical radiculopathy, hypothyroidism, hypertension, dyslipidemia, and latent tuberculosis infection, presented with severe GO (Figure 1). The patient was an active smoker and had a previous diagnosis of thyroid eye disease, complicating her overall clinical condition.

**Figure 2:**
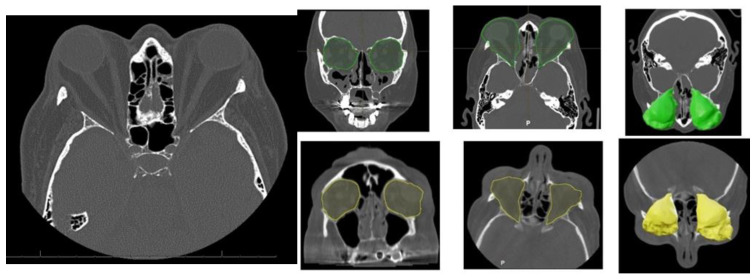
Characteristic orbital findings in Graves’ orbitopathy. Axial and coronal CT images showing hallmark features of Graves’ orbitopathy, including bilateral enlargement of extraocular muscles (notably the inferior and medial rectus), with sparing of the tendinous insertions. Increased orbital fat volume and anterior displacement of the globe (proptosis) are also visible. These changes contribute to compressive effects on the optic nerve and are commonly used for diagnostic and preoperative planning purposes. Preoperative intra-orbital volume shown in green; postoperative volume in yellow.

Orbital Computed Tomography (CT) imaging (Figure 2) revealed bilateral exophthalmos associated with substantial hypertrophy of intra- and extraconal orbital fat, alongside extraocular muscle enlargement. The superior recti muscles were primarily affected, with minor involvement of the right medial rectus and inferior recti muscles.

Preoperatively, the patient exhibited bilateral proptosis (24 mm in the right eye and 26 mm in the left eye), mild right-eye ptosis, and inferior eyelid retraction. Visual acuity was 0.7 in the right eye and 0.4–0.5 in the left due to amblyopia. Ocular motility was unrestricted, with no diplopia or reported pain. Bio-microscopy showed that chronic conjunctival hyperemia, and intraocular pressure was normal. The clinical activity score (CAS) was 0, indicating no active inflammation.

**Figure 3:**
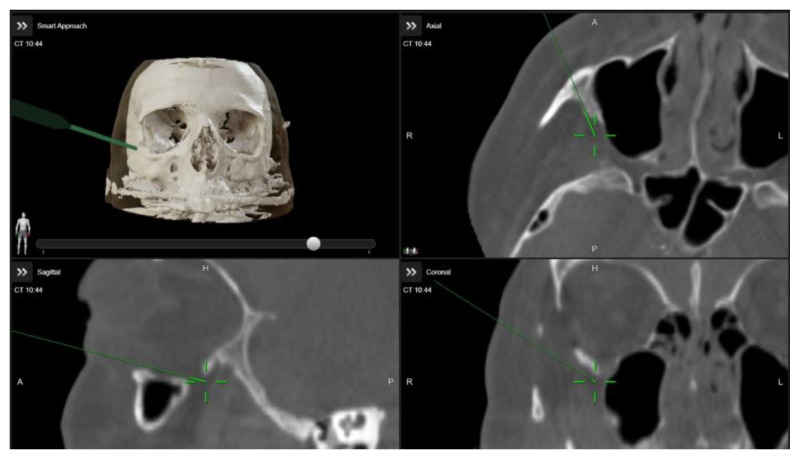
Intraoperative images demonstrating the use of image-guided navigation during orbital decompression surgery. The green arrow indicates the navigation pointer within the Brainlab system.

A multidisciplinary surgical approach was employed, involving craniomaxillofacial surgery, otolaryngology, and ophthalmology services. The procedure was planned to use computed tomography (CT) imaging with navigation markers. The images were processed using BrainLab^®^ software (Brainlab Elements, 2024 release), enabling precise multiplanar and three-dimensional (3D) anatomical assessment (Figure 3 and Video S1). Different orbital wall areas were color-coded to facilitate intraoperative identification.

**Figure 4:**
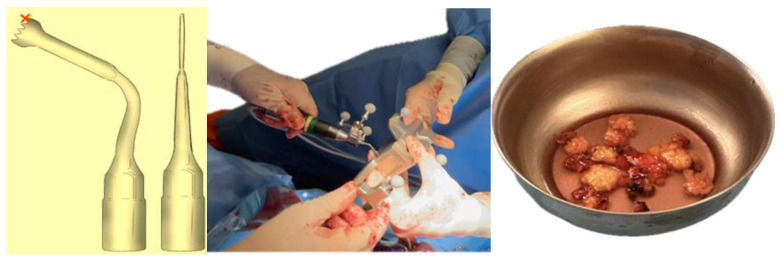
Cutting tip used in CANPS. Only the distal part of the instrument (marked with a red cross) is registered for surgical navigation, as it corresponds to the active cutting area. This is a standard design feature that provides sufficient accuracy for safe bone resection. The piezoelectric device is tracked using a clamped dynamic reference frame with three spheres and a calibrating matrix. Lipectomy of prolapsed orbital fat.

The choice of orbital decompression technique in Graves’ orbitopathy is guided by the severity of the disease and the degree of proptosis (Table 1). The surgical intervention comprised a two and a half-wall orbital decompression. Maxillofacial surgeons performed lateral wall and orbital floor bone resections via a combined transconjunctival and lateral approach on both sides. The procedure included lateral canthotomy and cantholysis, followed by pre-septal dissection to the orbital rim. Periosteal stripping facilitated lateral wall and orbital floor decompression. Simultaneously, otolaryngologists executed medial orbital wall decompression using a trans-nasal endoscopic approach. A dynamic reference frame affixed to a Mayfield support ensured patient stability, while virtual-to-real patient correlation was achieved through navigation pointer localization of three reference points. Bone resection was performed using computer-assisted navigated piezoelectric surgery (CANPS), and prolapsed orbital fat areas were excised (Figure 4).

Table 2 summarizes the orbital volume measurements obtained by computed tomography (CT) before and after surgical decompression in patients with Graves’ orbitopathy and compares them to established normative values. The mean normal orbital volume was 26.1 ± 3.3 cm^3^. In the affected orbits, preoperative volumes were already increased, averaging 30.1 cm^3^ in the right orbit and 30.3 cm^3^ in the left orbit. Following surgical decompression, the postoperative volumes further expanded to 33.6 cm^3^ and 34.3 cm^3^ in the right and left orbits, respectively. This corresponds to an average orbital volume increase of 3.5 cm^3^ in the right orbit and 4.0 cm^3^ in the left orbit.

These findings confirm that the surgical approach achieved substantial orbital expansion beyond physiological norms, reflecting the effectiveness of the decompression technique in relieving orbital pressure and accommodating proptotic content. Postoperatively, exophthalmos reduction was achieved (20 mm in the right eye and 21 mm in the left eye). Visual acuity decreased slightly (0.3 in the right eye and 0.4 in the left eye) related to transient postoperative ocular surface changes. Ocular motility evaluation revealed exotropia, with near vision at 18 prism diopters (pd) and distance vision exceeding 25 pd. A limitation in bilateral abduction was compensated by 18 pd in levoversion, with no limitations in elevation or depression. Binocular tests demonstrated positive fusion, orthophoria in the primary position, and no near vision suppression. Diplopia was significantly improved, now restricted to extreme right gaze.

**Figure 5:**
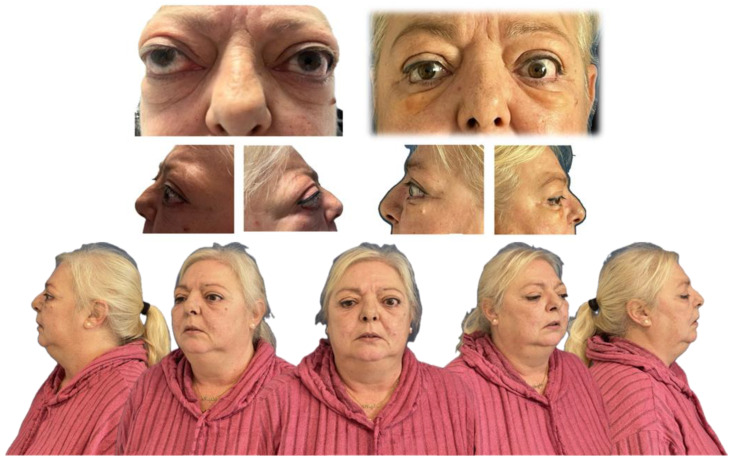
Preoperative and postoperative images demonstrating improvement in proptosis, eyelid retraction, and ocular alignment, with restoration of orbital contour and enhanced facial symmetry.

Orbital volumetric analysis was conducted using preoperative and postoperative CT scans, selecting axial orbital images for evaluation. BrainLab software facilitated orbital volume quantification, demonstrating a significant increase postoperatively (Table 2). These volume changes correlated with proptosis reduction and intraocular pressure normalization, underscoring the efficacy of orbital decompression. Postoperative outcomes demonstrated notable improvement in proptosis, eyelid position, and ocular motility, along with enhanced orbital contour and overall facial aesthetics (Figure 5).

## 3. Discussion

### 3.1. Clinical Assessment and Radiological Quantification in GO

Graves’ orbitopathy poses significant challenges due to pathological orbital tissue expansion, leading to proptosis and extraocular muscle dysfunction. Effective management requires precise quantification of volumetric changes [7,8,9]. Orbital decompression remains a cornerstone of treatment, with modern techniques shifting toward minimally invasive and targeted approaches that optimize both functional and aesthetic outcomes. To assess results, while the Clinical Activity Score (CAS) is effective for evaluating inflammatory activity, additional tools such as the GO-QoL questionnaire may offer complementary insights into patient-perceived quality of life and should be considered.

**Figure 6:**
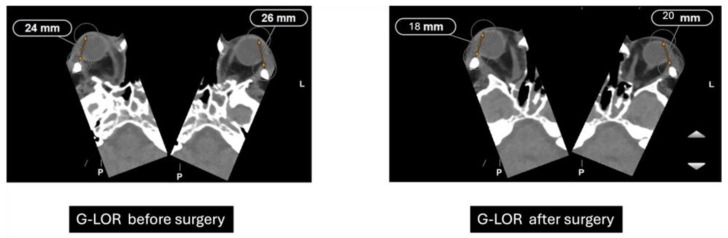
Preoperative and postoperative CT measurements of G-LOR (Globe-Lateral Orbital Rim) distance. Axial CT scans showing a reduction in proptosis following orbital decompression surgery. Preoperative G-LOR values measured 24 mm (**right**) and 26 mm (**left**), while postoperative measurements decreased to 18 mm (**right**) and 20 mm (**left**), indicating effective posterior repositioning of the globe.

Previously performed freehand and heavily dependent on surgeon experience, orbital decompression has evolved into a more predictable procedure through the integration of advanced imaging diagnostics and refined surgical techniques [10]. The Globe-Lateral Orbital Rim (G-LOR) distance is a widely accepted radiological measurement used to quantify proptosis in patients with Graves’ orbitopathy. This parameter, obtained from axial computed tomography (CT) scans, represents the linear distance between the anterior surface of the globe and the lateral orbital rim. Unlike clinical exophthalmometry, the G-LOR measurement provides high reproducibility and is not influenced by soft tissue swelling or examiner variability, making it especially useful for surgical planning and postoperative outcome evaluation (Figure 6) [11]. This CT-based measurement allows for precise preoperative assessment and postoperative comparison, particularly useful when planning or evaluating the efficacy of orbital decompression. Its reproducibility and correlation with clinical findings make it an important tool for both surgical decision-making and research standardization [12].

### 3.2. Image-Guided Surgery and Volumetric Outcomes

Image-guided surgery (IGS) has revolutionized orbital procedures by enhancing surgical precision and minimizing complications. At our institution, intraoperative navigation (BrainLab^®^) and intraoperative cone beam computed tomography (Loop-X^®^) have been adapted for orbital decompression, integrating preoperative imaging data with real-time intraoperative navigation. This system integrates preoperative imaging data, such as magnetic resonance imaging (MRI) or computed tomography (CT) scans, with real-time intraoperative feedback and allows surgeons to visualize the exact location of instruments relative to critical anatomical structures, optimizing surgical planning and execution [13].

Preoperative high-resolution imaging is used to generate a detailed three-dimensional (3D) model of the surgical site, which is uploaded to the Brainlab platform. Before surgery, we plan the surgical objective, designing the osteotomies and the resection of the orbital walls to achieve the final volumetric outcome. During surgery, specialized instruments equipped with sensors or optical markers interact with the system, allowing real-time tracking of their position and orientation relative to the preoperative model. This enables precise localization of surgical tools in relation to critical anatomical structures. By providing continuous feedback on instrument proximity to delicate regions, the system facilitates accurate decision-making and intraoperative adjustments, enhancing precision, optimizing surgical planning, and potentially reducing operative time and complications.

Introduction of image-guided surgery (IGS) has enhanced precision by enabling accurate localization of critical structures, facilitating more predictable osteotomy design, and improving control over intraconal volume expansion for proptosis correction. IGS achieves an average proptosis reduction of 3.8 mm, slightly surpassing outcomes from non-guided procedures. However, these advantages come at the cost of increased surgical time and expenses, underscoring the need for prospective studies to further assess its overall efficacy and long-term benefits [14]. The observed increase in orbital volume following surgical decompression reflects the efficacy of the procedure in creating additional intra-orbital space to alleviate proptosis in patients with Graves’ orbitopathy. In this cohort, postoperative volumes exceeded normal anatomical values by an average of 7.5 cm^3^ in the right orbit and 8.2 cm^3^ in the left orbit. These changes are consistent with previously published data indicating that orbital volume expansion of 3 to 5 cm^3^ is typically sufficient to achieve meaningful clinical improvement in proptosis and optic nerve decompression [5,13,15].

The greater-than-normal postoperative volumes suggest an aggressive but controlled decompression strategy, likely tailored to patients with significant proptosis or risk of optic neuropathy. This level of expansion, while effective in restoring orbital pressure balance, must also be weighed against the potential for postoperative complications such as new-onset diplopia, highlighting the need for individualized surgical planning based on preoperative volumetric analysis and clinical severity [6,16].

### 3.3. Imaging Technologies and Quantitative Analysis Tools

Quantifying orbital soft tissue volume is crucial in conditions such as GO. Various segmentation techniques, including manual and computer-assisted approaches, have been employed, yet many lack validation. Volumetric segmentation, widely utilized in radiotherapy, has evolved along with machine learning algorithms that enhance automation and reproducibility. In thyroid eye disease, deep learning has been applied to quantify orbital fat and muscle volumes, improving the understanding of disease pathophysiology, clinical features, and therapeutic responses. Some authors demonstrated the use of Mimics software to evaluate bone and fat volumes in patients with orbital fractures, showing consistency with other studies but without formal validation of the method. Commercial and open-source software often struggle to adapt to the irregular anatomical changes seen in thyroid eye disease, such as small orbital targets, irregular tissue expansion, and apical crowding [17]. Despite advancements, challenges remain. Manual segmentation remains labor-intensive, and commercial software often struggles to adapt to the irregular anatomical changes in GO. This study introduces BrainLab^®^ as a validated volumetric analysis tool, offering accurate, reproducible segmentation across diverse imaging modalities. While promising, BrainLab^®^ implementation requires specialized training and technological resources, which may limit accessibility in some clinical settings. Additionally, observer variability remains a concern, reinforcing the need for standardization and training [16].

### 3.4. Surgical Strategies and Future Directions

Recent advancements, like the “temporal cage”, have improved both functional and aesthetic outcomes by supporting orbital tissue redistribution. The comparison between open and endoscopic trans-nasal decompression techniques demonstrates comparable proptosis reduction, with endoscopic methods offering superior cosmetic and less invasiveness, making it a preferred option for direct access to the orbital apex in complex cases. The choice between techniques should consider patient-specific factors and surgeon expertise, underscoring the need for studies evaluating long-term outcomes [17].

GO management typically follows a staged approach, beginning with orbital decompression, followed by extraocular muscle correction and eyelid repositioning [18,19]. Combined interventions may reduce the number of surgeries required, improving patient outcomes. However, the scarcity of randomized controlled trials and long-term follow-up studies hinders protocol standardization. Future research should focus on validating these techniques in larger, diverse patient cohorts and integrating advanced imaging and biomarkers into personalized treatment strategies.

In summary, Graves’ orbitopathy necessitates multidisciplinary and individualized surgical management. Advances in biomarkers, computer-assisted technologies, and innovative surgical techniques have transformed treatment, enhancing functional and aesthetic outcomes. Despite these advancements, further refinement is required to optimize benefits and minimize complications, ultimately improving patients’ quality of life.

## 4. Conclusions

This case underscores the critical importance of a multidisciplinary approach and advanced surgical technologies in the management of severe Graves’ orbitopathy. Image-guided orbital decompression allowed for precise osteotomy planning, real-time intraoperative navigation, and a planned intraconal volume expansion, resulting in measurable proptosis reduction and clinical improvement. The integration of computer-assisted planning and intraoperative navigation systems enhanced surgical predictability and safety, minimizing risks associated with traditional techniques. Furthermore, close collaboration among maxillofacial surgeons, otolaryngologists, and ophthalmologists was pivotal to achieving optimal functional and aesthetic outcomes. These findings support the adoption of image-guided surgery as a standard of care in complex orbital procedures and highlight the necessity of coordinated multidisciplinary treatment in achieving long-term therapeutic success in Graves’ orbitopathy.

## Figures and Tables

**Table 1:** Treatment algorithm for orbital decompression in Graves’ orbitopathy according to severity.

Severity Level	Proptosis	Surgical Technique	Expected Improvement
🟢 Grade 1: Mild	<24 mm	Orbital fat resection; conservative approach. Eyelid surgery if needed.	Variable
🟡 Grade 2: Moderate without neuropathy	24–27 mm	Lateral wall decompression ± balanced medial wall decompression.	≈4.8 mm
🟠 Grade 3: Moderate with neuropathy	24–27 mm (Neuropathy)	“One-and-a-half wall” technique: medial wall, posterior-medial floor decompression, optic nerve decompression.	2.7–4 mm
🔴 Grade 4: Severe	>27 mm	“Two-and-a-half walls” technique: medial, lateral walls and posterior-medial floor decompression.	Significant
⚫️ Grade 5: Refractory cases	Persistent severe proptosis, no response to previous surgery	Additional anterior orbital floor decompression.	Additional improvement

**Table 2:** Orbital volume analysis in Graves’ disease: preoperative, postoperative, and volume change after surgical remodeling compared to normal reference.

Orbit	Normal (cm^3^)	Preoperative (cm^3^)	Postoperative (cm^3^)	Volume Increase (cm^3^)
Rigth orbit	26.1 ± 3.3	30.1	33.6	3.5
Left orbit	26.1 ± 3.3	30.3	34.3	4.0

Normal orbital volumes presented as mean ± standard deviation (SD). Volumes measured using computed tomography (CT).

## Data Availability

The data presented in this study are available on request from the corresponding author.

## Supplementary Materials

The following supporting information can be downloaded at: https://www.mdpi.com/article/10.3390/life15081277/s1, Video S1: Volumetric Analysis of Navigation-Guided Orbital Decom-pression in Graves’ Orbitopathy.

## Abbreviations

The following abbreviations are used in this manuscript:

GO	Graves’ orbitopathy
IGS	Image-guided surgery
CT	Computed Tomography
CAS	Clinical Activity Score
G-LOR	Globe-Lateral Orbital Rim
CANPS	Computer-assisted navigated piezoelectric surgery
